# Universal access to essential medicines as part of the right to health: a cross-national comparison of national laws, medicines policies, and health system indicators

**DOI:** 10.1080/16549716.2019.1699342

**Published:** 2020-11-02

**Authors:** Katrina Perehudoff

**Affiliations:** a Social & Behavioural Health Sciences Division, Dalla Lana School of Public Health, University of Toronto, Toronto, Canada; b International Centre for Reproductive Health- WHO Collaborating Centre, Department of Public Health & Primary Care, Ghent University, Ghent, Belgium

**Keywords:** Access to medicines, human rights, pharmaceutical policy, universal health coverage, national medicines policy, essential drugs, health financing, vulnerable populations, constitution, litigation

## Abstract

**Background:**

Access to essential medicines for the world’s poor and vulnerable has made little progress since 2000, except for a few specific medicines such as antiretrovirals for HIV/AIDS. Human rights principles written into national law can create a supportive environment for universal access to medicines; however, systematic research and policy guidance on this topic is lacking.

**Objective:**

To examine how international human rights law and WHO’s essential medicines policies are embedded in national law for medicines affordability and financing, and interpreted and implemented in practice to promote universal access to essential medicines.

**Methods:**

This thesis consists of (1) a cross-national content analysis of 192 national constitutions, 71 national medicines policies, and legislation for universal health coverage (UHC) from 16 mostly low- and middle-income countries; (2) a case study of medicines litigation in Uruguay, and (3) a follow-up report of eight right to health indicators for access to medicines from 195 countries.

**Results:**

Some, but not all, of the 12 principles from human rights law and WHO’s policy are embedded in national UHC law and medicines policies (part 1). Even the most rights-compliant legislation for access to medicines is subject to the unique and inconsistent interpretation of domestic courts, which may be inconsistent with the right to health in international law (part 2). Many national health systems for which data were available still fail to meet the official targets for eight indicators of access to medicines (part 3).

**Conclusions:**

International human rights law and WHO policy are embedded in national law for essential medicines and practically implemented in national health systems. Law makers can use these findings and the example texts in this thesis as a starting point for writing and monitoring governments’ rights-based legal commitments for access to medicines. Future research should study the effect of national law on access to medicines and population health.

## Background

This is a PhD Review that provides a synthesis of my doctoral thesis titled ‘The right to health as the basis for universal access to essential medicines: A normative framework and practical examples for national law and policy’. An estimated 400 million people do not receive essential health services, including vaccines and medicines for modern family planning methods, antiretroviral therapy for HIV, and tuberculosis treatment. [1] Striking regional disparities exist for several of these basic health services: coverage rates are lowest in Sub-Saharan African and South Asian countries, where one-third of the world’s population lives. (1)⁠ The Lancet Commission on Essential Medicines Policies identified five key barriers to universal access to essential medicines: medicines affordability, sustainable financing, medicines quality, optimal medicines use, and research and development of needed medicines. (2)⁠ This article focuses on medicines affordability and financing. Essential medicines are defined by the World Health Organization (WHO) as ‘those that satisfy the priority healthcare needs of the population’. (3)⁠ Essential medicines are chosen considering the local disease prevalence, efficacy, safety, and comparative cost effectiveness [2].

Low- and middle-income countries (LMICs) often have a large proportion of financially-vulnerable people who are dependent on government-subsidised essential medicines provided at public health facilities without charge or for a nominal fee [3,4]. However, frequent stock-outs in these facilities force patients to turn to the private sector, where medicines are available but often at a higher price [3,4]. For example, as much as 60% of households in low-income, 33% in lower-middle, and 25% in upper-middle income countries could not afford four commonly used cardiovascular medicines sold in private pharmacies [5]. Unaffordable medicines confront households with potentially catastrophic health spending, or force families to forgo treatment at the expense of their health and possibly their livelihood [3,6]. Despite high-level political commitments to improving the affordability of medicines (i.e. for non-communicable diseases), access to essential medicines for the world’s poor has made little progress, except for a few medicines such as antiretrovirals [7,8].

To address these challenges, universal health coverage (UHC) is an initiative to broaden equitable access to financial protection and quality essential health care such that ‘all people have access to needed health services (including prevention, promotion, treatment, rehabilitation and palliation) of sufficient quality to be effective while also ensuring that the use of these services does not expose the user to financial hardship’ [9]. UHC offers financial risk protection to all, including low-income households, by raising funds from pre-paid insurance (and sometimes government contributions) and reducing households’ reliance on out-of-pocket expenditures [1]. As such, UHC is an important means for governments to make quality essential medicines available, accessible, and affordable to the vulnerable populations, particularly in LMICs, who currently lack access. Universal access to essential medicines is an integral part of the right to health and UHC, reflected in the Sustainable Development Goal (SDG) 3 for health and SDG Target 3.8 [10].

### Essential medicines as part of the right to health

Human rights have the potential to transform social, political, and legal norms for more equitable access to medicines [11]. The right to health is legally binding on the 169 national governments that have ratified the International Covenant on Economic, Social, and Cultural Rights (ICESCR). Consequently, these governments are legally obliged to protect and promote health rights in national law (a term I use to convey ‘domestic law’, meaning all law made by all levels of a government) and policy. The strength of each country’s compliance varies depending on the standing of international law in the domestic legal order, and on the presence and content of national implementing legislation. [12]. In 2000, General Comment No. 14, which is an authoritative interpretation of the right to health by the UN Committee on Economic, Social, and Cultural Rights, established that States partyhave the minimum ‘core obligation’ to provide essential medicines, defined by WHO, with a maximum of available resources [13]. Core obligations are basic minimum standards that serve as the foundation of all other aspects of the right to health [14]. Legal provisions in national law that include right to health language can create a supportive environment for poor patients to claim government -subsidised essential medicines [15,16]. (‘Legal provisions’ mean the legal language that is used to articulate underlying principles for access to medicines.)

### National law as an intervention to promote access to medicines

National law is a powerful intervention that can promote equitable access to health services and financial coverage for the most vulnerable people [17]. WHO’s David Clarke and colleagues explain that ‘a strong legal framework sets the rules for how the health system functions, establishes a legal mandate for access to health services and provides the means by which a national government can implement universal health coverage at a population level’ [17]. Indeed, national governments frequently use legal tools to shape health systems in response to their available resources and public health needs. Between 2011 and 2014, 70 countries sought WHO’s advice for scaling up UHC [18].

Currently, little research investigates whether and how national law supports universal access to essential medicines as part of UHC. The first challenge is the absence of a reliable and up-to-date global repository of national health law. There are two pilot studies from 2010 of language supporting access to medicines in national constitutions and national legislation [19,20]. These studies were an important first step to explore national law and medicines provision; however, the conclusions are limited by shortcomings in the search strategy, the small sample of 4 countries, and little recognition for the then-novel UHC concept. In 2017 WHO and its partners published the report *Advancing the right to health: Vital role of the law*, offering general legal guidance for Member States on a variety of public health laws, yet none comprehensively cover access to medicines [21]. In 2018, WHO Europe published a collection of medicines reimbursement policies with nine country case studies [22]. Although these are insightful descriptive comparisons of policies in practice, this document lacks a multi-dimensional critical analysis, including from the perspective of human rights [22]. This paucity of evidence and policy advice illustrates how emergent such legal studies are in the field of pharmaceutical policy research.

In addition, little is known about to what degree national governments realise their human rights obligations to provide essential medicines to those who can not provide for themselves. A human rights approach can employ indicators to monitor changes in health systems. Three global initiatives have sought to identify and, where possible, collect access to medicines indicators; however, none of these initiatives offer are up-to-date measures of access to medicines as a part of the right to health. In 2008, the then-UN Special Rapporteur on the Right to Health reported on 72 right to health indicators (including eight indicators of access to medicines) in 194 health systems. These indicators have not been updated since 2008 [23]. The Millennium Development Goals (MDGs) and the SDGs provide robust data on national measles immunisation rates from many countries while reporting on essential medicines availability from very few countries [24,25,26]. Drawing from the pool of all foregoing indicators, the Lancet Commission on Essential Medicines Policies proposed a set of indicators (published during the completion of this thesis) and later supported a call for a global accountability mechanism for monitoring access to essential medicines [3,27].

### Aims

The question guiding this thesis is: *How has international human rights law and WHO’s essential medicines policy has been embedded in national law and policy for medicines affordability and financing, and been interpreted and implemented in practice to promote universal access to essential medicines?* This thesis aims to offer first-ever insight into how access to medicines is framed in the legal provisions of national law and policy,and to explore tracer indicators that signal related impacts on access to medicines in health systems. ‘National law’ is understood to encompass the written rules adopted by government institutions and agencies that impose both legally binding legislation, whereas ‘policies’ are the non-binding documents or ‘soft’ policies, strategies, or plans of action.

The central premise of this thesis is that national legal frameworks that include principles and language from international human rights law and WHO’s essential medicines policies, can remedy the widespread political indifference in attaining universal access to essential medicines. This thesis focuses on laws and policies that could secure access to medicines for poor people in mostly low- and middle-income countries.

The specific aims of this thesis are:

1. to collect and critically analyse the content of different types of national laws and policies (‘legal architecture’) related to medicines affordability and financing, against the global standards established in international human rights law and WHO’s essential medicines policies;
2. to investigate how robust national laws promoting universal access to medicines (identified in aim 2) are interpreted in light of international human rights law by national courts in judicial decisions (‘lawmaking’);
3. to understand how national governments realise their right to health obligations for essential medicines by updating the 2008 UN Special Rapporteur on the Right to Health’s report on eight right to health indicators of access to medicines in 195 health systems (health ‘environment’).

### Conceptual framework

**Figure 1.**
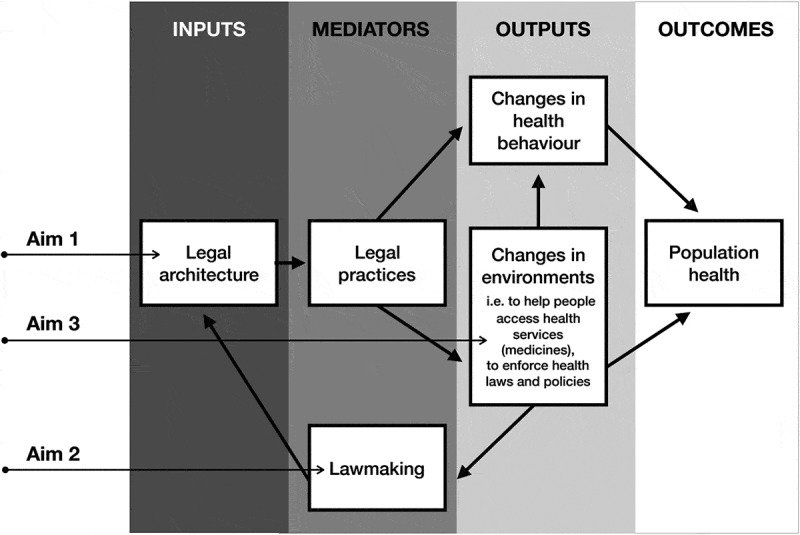
Model of the effect of national law on access to medicines and population health, modified from Scott Burris and Alexander Wagnaar. The objectives of this thesis are situated at aims 1, 2, and 3 in the model.

To illustrate why national law and policy, and health systems indicators are relevant for understanding access to medicines and related health outcomes, this thesis employs a modified version of Scott Burris and colleagues’ model to study the effect of national law and policy on population health [28,29]. See Figure 1. In this model *legal architecture* refers to national legislation and policy, understood to be the written and unwritten rules that can impact on public health. In the context of access to medicines, ‘legal architecture’ may include domestic constitutions, legislation and regulation for universal health coverage and public health, and policies (i.e. national medicines policy (NMP), national health strategy or plan of action, etc.).

The mediators in the model are the *legal practices* of actors (i.e. healthcare professionals, police, etc.) and institutions that give laws meaning and translate them into practice. For example, legal practices are those actions of physicians who give effect to generic substitution laws or policies by prescribing medicines by international non-proprietary name.

*Legal practices* can lead to two types of outputs: changes in health system performance, or the *environment*, and changes in an individual or a population’s *health behaviour*. The *environment* refers to the physical surroundings, social structures, and institutions related to health, which are affected by fluctuations in the available resources (ex. public funding for the provision of essential medicines can increase access to medicines), rights and obligations, and incentives and penalties (ex. incentives for pharmacies to dispense generic medicines to patients) [29]. Changes in the health environment aim to achieve a number of goals, which, for essential medicines, include helping people access medicines, and/or enforcing medicines-related laws and policies [29]. *Health behaviour*. is either directly affected by legal practices or indirectly through changed environments, both of which make particular behaviours more or less attractive. For example, legislation that changes the health environment by requiring the government to subsidise the cost of essential medicines for patients, could consequently also increase patient demand for these medicines, changing their health behaviour.

A feedback loop exists where changes in the environment influence *lawmaking*, known as the activities of legal actors (i.e. legislative and judicial branches, policy makers in other areas of the health system) that result in written and unwritten rules. Lawmaking includes the actors and the factors that determine which laws are adopted, and the characteristics and interpretation of those laws [29]. For example, national law (the *legal architecture*) requires the provision of government-funded essential medicines (the *health environment*), which may increase patient demand for those and other non-essential medicines (*health behaviour*). In Latin America, patients turn to the courts to request publicly-funded medicines that are not currently accessible/affordable. In these cases, the domestic court interprets the original domestic law in light of constitutional rights and obligations to determine if the patient’s request for medicines should be granted, which is the process of *lawmaking*.

The key outcomes in this model are changes in *population health*, such as rates of morbidity or mortality.

## Methods

This thesis is a multidisciplinary inquiry drawing from the legal and health science disciplines. This synthesis consists of three parts: (1) qualitative document analysis of different types of national laws and national medicines policies, (2) a case study of lawmaking in Uruguay, and (3) a quantitative report of right to health indicators for access to medicines from 195 countries. Methodological aspects of these three parts are summarised in Table 1.

**Table 1.** Overview of studies and methodologies in this article.

	Part 1: ‘Legal architecture’ for access to medicines	Part 2: ‘Lawmaking’ for access to medicines through Uruguayan courts	Part 3: Access to medicines indicators in the health system ‘environment’
Study design	Qualitative document analysis	Case study	Quantitative descriptive report
Method of analysis	Cross-sectional comparative content analysis of different types of national law	Content analysis of Uruguayan *amparo* decisions about access to medicines	Cross-sectional follow-up report of access to medicines indicators
Framework of analysis	a) Right to health in the ICESCR and General Comment No. 14, specifically the government duties to respect, protect, and fulfil the provision of essential medicines b-c) 12-point policy checklist derived from international human rights law and WHO’s essential medicines policies [38,14] described in Table 2	Right to health in the ICESCR and General Comment No. 14, specifically the core obligations to provide essential medicines on a non-discriminatory basis	Right to health in the ICESCR and General Comment No. 14, specifically eight indicators described in Table 3
Data (sources)	Primary: a) National constitutions [52] b) National medicines policies (structured online search described in [3]) c) National legislation for universal health coverage (structured online search described in [38])	Primary: *Amparo* decisions made by the 7 national appeals circuits about pharmaceutical therapies [30]	Primary: a) National constitutions [52] b) National medicines policies (structured online search described in [3]) c) National essential medicines policies (structured online search described in [3]) Secondary: d) Public per capita expenditure on pharmaceuticals [31,32,33] e-f) Availability of a basket of essential medicines in the public and private sectors [34] g) Proportion of children who had received two doses of a measles virus-containing vaccine (MCV2) [35] h) Proportion of children who had received three doses of a vaccine for diphtheria-tetanus-pertussis (DTP3) [36]
Method of selecting countries	a-b) All retrievable data from all countries c) Purposive sample of 16 countries. See selection criteria.	See selection criteria.	a-h) All retrievable data from all countries
Number of countries	a) 192 countries b) 71 countries c) 16 countries	Single country (Uruguay)	a) 192 countries e) 28 countries b) 157 countries f) 30 countries c) 173 countries g) 153 countries d) 70 countries h) 98 countries
Years covered in data analysis	a) 2015 b) 1990–2016 c) 1961-2016	2015	a) 2015 e) 2008–2015 b) 1990–2016 f) 2008–2015 c) 1998–2015 g) 2015 d) 2008–2013 h) 2015

**Table 2.** 12-point policy checklist for access to essential medicines applied to in national law. Data sources: 50–51.

Checklist	Strong provisions in national medicines policies (n countries/71 countries)	Strong provisions in legislation for universal health coverage (n countries/16 countries)	Countries with strong provisions in UHC legislation for medicines affordability and financing for vulnerable groups
1. Right to health	13/71 countries (18.3%)	9/16 countries (56.3%)	Text includes a universal entitlement to health coverage includes medicines: Colombia, Chile, Ghana, Indonesia, Mexico, Nigeria, Tunisia, Turkey, Uruguay
2. State obligation to provide essential medicines	17/71 countries (23.9%)	8/16 countries (50.0%)	Text includes an absolute State obligation to realise or guarantee UHC and (affordable) access to medicines: Colombia, Chile, Ghana, Indonesia, Mexico, Philippines, South Africa, Uruguay
3. Transparency	19/71 countries (26.8%)	3/16 countries (19.8%)	Text requires that information or transparency about medicines affordability and accessibility be available to patients: Chile, Philippines, South Africa
4. Participation & consultation	2/71 countries (2.8%)	3/16 countries (19.8%)	Text includes the principle of and a mechanism for the participation or consultation of patients or users in medicines policies: Chile, Colombia, Mexico
5. Monitoring & evaluation	15/71 countries (21.1%)	2/16 countries (12.5%)	Text requires the State to monitor the affordability and/or accessibility of medicines for users with UHC: Mexico, Philippines
6. Accountability & redress	0/71 countries (0%)	9/16 countries (56.3%)	Text includes the principle of or right to accountability as well as a non-judicial mechanism for patients to make complaints or seek redress: Algeria, Chile, Indonesia, Mexico, Nigeria, Philippines, Rwanda, South Africa, Turkey
7. Selection of essential medicines	44/71 countries (62.0%)	7/16 countries (43.8%)	Text includes the principle of and a mechanism for (essential) medicines selection: Chile, Colombia, Ghana, Mexico, Nigeria, Indonesia, Uruguay
8. Government financing	24/71 countries (33.8%)	5/16 countries (31.3%)	Text includes a clear State obligation to finance essential medicines or medicines in a UHC benefits package: Chile, Colombia, Mexico, Nigeria, Philippines, Turkey
9. Pool user contributions	5/71 countries (7.0%)	11/16 countries (68.8%)	Text requires the compulsory pre-payment of UHC contributions with exceptions for those who can not pay: Colombia, Chile, Ghana, Indonesia, Jordan, Mexico, Morocco, Philippines, Rwanda, Tunisia, Turkey
10. International assistance and technical cooperation	12/71 countries (16.9%)	1/16 countries (6.3%)	Text requires that the State seek financial aid and/or technical assistance from the international community: Mexico
11. Efficient and cost-effective spending	43/71 countries (60.6%)	7/16 countries (43.8%)	Text includes the principle of cost-effectiveness and/or efficiency, as well as one or more mechanisms applying these principles to medicines (i.e. health technology assessment): Colombia, Chile, Indonesia, Mexico, Philippines, Turkey, Uruguay
12. Financial protection of vulnerable groups	17/71 countries (23.9%)	9/16 countries (56.3%)	Text includes a clear State duty to finance a UHC package and/or essential medicines for vulnerable people: Chile, Colombia, Ghana, Indonesia, Jordan, Mexico, Philippines, South Africa, Uruguay

### Analytical frameworks

This thesis derives its analytical framework from the right to health as it is conceptualised in the ICESCR (article 12) and elaborated in General Comment No. 14. General Comment No. 14 is an authoritative interpretation of the right to health in the ICESCR; it is a non-binding document that instructs States on which aims and actions will realise their legal obligations in the ICESCR.

Part 1 applies two analytical frameworks that are appropriate to the scope, content, and detail of the different types of law under investigation. Constitutions are analysed through the lens of the tripartite typology, where the State has a duty to *respect, protect*, and *fulfil* access to medicines as part of the right to health [13,37]. *Respect* and *protect* are negative duties to refrain from interference and to safeguard individuals from the actions of third parties, respectively. *Fulfil* is a positive duty to take steps to ‘progressively realise’ the right to health, such as through the provision of health facilities, goods, and services. Progressive realisation is an obligation to take deliberate, targeted, and concrete steps towards realising the right to health.

The detail of national medicines policies and UHC legislation in part 1 lends itself to a deeper analyses using the 12-point policy checklist previously developed by the author and three colleagues as part of this thesis [38,14]. See Table 2. This policy checklist is based on overlapping principles in WHO’s policies for essential medicines and international human rights law that are important for access to medicines. Principles were drawn from WHO’s essential medicines policies, specifically for medicines affordability and the financial protection of vulnerable groups [39,40,41,42,43], and from international human rights law in relation to States’ obligations towards social or health rights, the core obligation to provide essential medicines, and/or rights related to good governance [12,40,41,42,43,44,45,46,47]. The normative development of this 12-point policy checklist is described elsewhere [38,14]. It serves as a global normative framework that is both a checklist and a wish list to evaluate State action to provide essential medicines.

In part 2, the analytical framework applies two core obligations under the right to health: the State duty to provide essential medicines, and the State duty to ensure access to health goods (including but not limited to diagnostics, devices, technologies, and medicines, which are the focus of this article) on a non-discriminatory basis [13,48].

In part 3, the analytical framework matches right to health principles with corresponding public health data to gauge the realisation of human rights standards in a population [49]. Each indicator corresponds to a specific State obligation under the right to health and has a global target, usually established by WHO. See Table 3.

**Table 3.** Eight right to health indicators of access to medicines. Data source: 48.

Type of indicator	Right to health indicator [23]	Human rights principle in General Comment No. 14 [13]	Global target
Structural	1. Constitutional commitment to medicines	Legal obligation to realise health rights	Medicines recognised in national constitutions [50]
	2. National medicines policy	Duty to adopt a national health plan	National medicines policy is adopted [39]
Process	3. National essential medicines list	Duty to adopt appropriate administrative measures to a maximum of its available resources. (Assured) quality of health services (of the AAAQ)	National essential medicines list is adopted [50]
	4. Government spending on pharmaceuticals	Financial accessibility of health services (of the AAAQ)	US$ 12.90–25.40 per capita per year [3]
Outcome	5. Essential medicines availability in the public sector	Availability of health services (of the AAAQ)	80% average national availability in both sectors [50]
	6. Essential medicines availability in the private sector		
	7. National child immunisation rate for measles	Duty towards non-discrimination and attention to the vulnerable	95% coverage with a measles-containing vaccine to eradicate disease [51]
	8. National child immunisation rate for the third dose of DTP		90% coverage of 3 doses of DTP vaccine to eradicate disease [50,51]

This table is derived in part from an article published in Global Public Health, 6 September 2018, copyright Taylor & Francis available online at https://doi.org/10.1080/17441692.2018.1515237.

Abbreviations used in this table: AAAQ = Availability, Accessibility, Acceptability, and Quality as elements of health services under the right to health.

### Part 1: ‘legal architecture’ for access to medicines

Part 1 is a mapping study of national law and policy, and cross-national comparative content analysis of legal provisions related to medicines. The subject of study is written, formalised national law, which includes all available constitutions and UHC legislation related to medicines from a purposive sample of 16 mostly LMICs (Algeria, Chile, Colombia, Ghana, Indonesia, Jordan, Mexico, Morocco, Nigeria, Philippines, Rwanda, South Africa, Tanzania, Turkey, Tunisia, Uruguay), as well as all retrievable full-text national medicines policies adopted by 2015. The 16 countries each have a universal health coverage scheme (at an early, intermediate, or advanced stage), have ratified the ICESCR, and represent a diversity of legal traditions, income economies and world regions. The country selection was also in function of the research assistants’ language capabilities and the presence of binding, federal UHC legislation. This explains why the selection does not include any of the ‘BRICS’ nations (Brazil-Russia-India-China-South Africa) except South Africa. Although not representative of all LMICs with UHC, other countries should be able to learn from the examples of a comparable country in this sample.

This study also compared the number and strength of the 12 principles in national medicines policies adopted before (n = 32) and after (n = 39) January 1^st^, 2004. The year 2004 was selected as a cut-off year because WHO’s latest guidance document for developing a national medicines policy (2^nd^ edition) was published in 2001 and the author estimated a lag-time of two years (i.e. 2002–2003) would be reasonable for national governments to introduce principles from the 2001 document into the content of subsequent national policies. Associations were determined in SPSS version 25 using Pearson’s Chi-squared statistic with significance of p < 0.05.

English-language translations of constitutions were sourced from the Constitute Project [52]. In the absence of a global repository of national health law, a structured online search (i.e. mapping exercise) was used. Electronic copies of the NMPs and UHC laws in the original language were crowdsourced. Multilingual researcher assistants – often working in multidisciplinary teams from the Faculties of Medical Sciences and Law – compiled in-depth, descriptive country profiles using a template that detailed the national demographics, health system structure and function, and legislation in relation to UHC and access to medicines. Research assistants fluent in the national language produced unofficial translations of legislation and policy when official English translations were not available. Translations for UHC law were peer reviewed by a second research assistant, except for Jordan and Turkey. At least one local pharmaceutical policy expert per country was consulted to verify the accuracy, relevance, and completeness of the primary sources and country profiles. No peer reviewers were located from Algeria or Nigeria in the author’s and supervisors’ broader network of access to medicines professionals.

Legal provisions were located by using explicit search terms and through a manual search of each document. Constitutional text was identified, categorised using the abbreviated framework of analysis, and reviewed by two pharmaceutical policy and human rights experts. For national policy and UHC legislation, two researchers coded the excerpts from national medicines policies and UHC legislation using explicit definitions defined in the 12-point policy checklist. The researchers deliberated any coding differences until consensus was reached.

### Part 2: ‘lawmaking’ for access to medicines through uruguayan courts

Part 2 is a single-country case study of lawmaking by the Uruguayan appeals courts. It critically analyses whether and how human rights arguments are used by the Uruguayan judiciary in relation to patients’ claims for access to specific publicly-financed medicines. Uruguay was selected as a case study because the State has ratified the ICESCR, it has equity-based UHC legislation that includes a State obligation to provide and an individual right to access medicines on a positive reimbursement list (determined in part 1), and the right to health is justiciable in national courts.

All retrievable *writ of amparo* cases claiming a pharmaceutical intervention and decided in 2015 were included in this study. A *writ of amparo* is a judicial procedure that individuals can use to claim that their fundamental constitutional right(s) are at immediate and significant risk. These procedures usually need to be decided within one week of filing [48]. This selection offers a snapshot of legal interpretation by the courts at the peak of medicines litigation in Uruguay and in the period immediately following legal reform designed to curb medicines litigation.

Cases were retrieved from the official Uruguayan national judiciary online databank (keywords ‘acceso’ and ‘medicamento’). Key facts of each case were extracted for analysis, including the facts of the case (i.e. medicine claimed, indication, reimbursement status, etc.), relevant laws and rights invoked in the case, and the legal arguments in the court’s decision.

### Part 3: access to medicines indicators in the health system ‘environment’

Part 3 is a follow-up report of the eight right to health indicators that were chosen by the UN Special Rapporteur on the Right to Health to reflect access to essential medicines, from 195 countries [23]. See the eight right to health indicators in Table 3. In addition, this study examines the feasibility of using these indicators as dependent variables to evaluate the impact of national law and policy.

Data for 195 countries was collected through systematic online searches and authoritative secondary online datasets. This article compares median achievements of countries grouped by income economy (World Bank definition) on each indicator to the established global targets (see Table 3). Country-level achievements on all eight indicators and historical trends (between the original 2008 report and this follow-up report) are reported elsewhere [53].

## Results

### Part 1: ‘legal architecture’ for access to medicines

Example texts from national law and national medicines policies that express human rights duties to provide essential medicines in legal language are provided elsewhere [37,38].

#### Constitutions

Twenty-two constitutions included the *duty to protect* and/or *to fulfil* access to essential medicines; these commitments were not mutually exclusive. The *duty to protect* requires the State to safeguard individuals from possible deleterious actions of third parties. In the case of medicines, 14/192 national constitutions required governments to regulate pharmaceuticals or monitor their quality, and/or ensure that access to medicines is not restricted by international trade agreements and commercial rights. Thirteen of 192 national constitutions embed a State obligations to provide medicines, vaccinations, and/or essential goods (*duty to fulfil*) as part of the right to health. No constitution obliges the State *to respect* the provision of essential medicines (i.e. Respecting essential medicines could entail the government not interfering with access to specific classes of medicines such as contraception or for medical abortion) [54]. Full results are reported elsewhere [37].

#### National medicines policies

**Figure 2.**
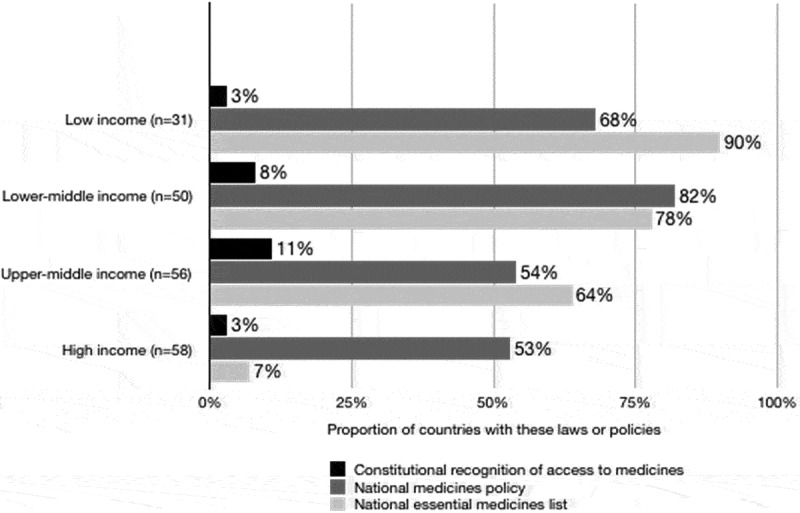
Proportion of countries with a constitution that recognises access to medicines, a national medicines policy, or a national essential medicines list.

Seventy-one full-text national medicines policies were retrieved. See Figure 2. Of the 12 principles, those of *essential medicines selection* (62.0% of national medicines policies) and e*fficient spending/cost-effectiveness* (60.6%) were most frequently embedded in policies. See Table 2. *Pooling user contributions* (7.0% of national medicines policies), *participation of beneficiaries* (2.8%), and *accountability and redress* (0%) were infrequent in many policies. Commitments to medicines affordability and financing are strongest in the policies of South Africa (1996), Suriname (2005), the Philippines (2011–2016), El Salvador (2011), and Somalia (2013).

Some principles that appeared for first time in the second edition of WHO’s guidelines for medicine policies (published in 2001), are significantly more frequent in national medicines policies adopted in 2004 or later. These principles are: the *right to health* (n_≤2003_, = 0/32 policies, n_≥2004_ = 13/39 policies, p = 0.000), *government financing* (n_≤2003_, = 6/32 policies, n_≥2004_ = 18/39 policies, p = 0.015), *efficient spending/cost-effectiveness* (n_≤2003_, = 14/32 policies, n_≥2004_ = 29/39 policies, p = 0.009), and *financial protection of vulnerable populations* (n_≤2003_, = 4/32 policies, n_≥2004_ = 13/39 policies, p = 0.041). Full results are reported elsewhere [55].

#### National legislation for universal health coverage

One hundred UHC and medicines-related laws were retrieved for 16 countries. The principles most frequently legalised in national law were *pooling user contributions* (in legislation from 68.8% of countries studied), *accountability* (in 56.3%), the *right to health* (56.3%), *financial protection of vulnerable populations* (56.3%), *State obligation* (50.0%). See Table 2. The least common principles were for *transparency* (in legislation from 19.8% of countries studied), *participation* (19.8%), *monitoring* (12.5%), and *international assistance and cooperation* (6.3%). Overall, UHC legislation from Colombia, Chile, Mexico, and the Philippines codifies the most principles for access to medicines.

Three trends for access to medicines were more common, although not significant, in the legislation of upper-middle and high income countries than the low- and lower-middle income countries samples. These trends are: (a) affluent countries (i.e. upper-middle and high income countries) embed explicit individual rights and state obligations about medicines in national law; (b) affluent countries establish in law clear boundaries to these entitlements and obligations; (c) affluent countries codify mechanisms for accountability and redress in national law. A full explanation of these observations is available elsewhere [56]. These trends generate hypotheses to be tested in a larger sample of countries.

### Part 2: ‘lawmaking’ for access to medicines through Uruguayan courts

Of the 42 claims included in this study, 31 (74%) were decided in favour of the claimant (i.e. usually the patient); 34 claims (81%) accounted for 10 medicines; eight claims (19%) successfully acquired the non-reimbursed medicines cetuximab, lenalidomide, and sorafenib. Interestingly, these medicines were explicitly excluded from the national medicines formulary by Ministerial Order 86/2015 because they are cost-ineffective for their indications. Complete results are reported elsewhere [48].

In the judicial decisions in this sample, the court inconsistently interpreted patients’ rights and the State’s legal obligations in line with the right to health. Two similar claims for cetuximab for the treatment of metastatic colon cancer illustrate this inconsistency. In the first case, Appeals Circuit/Court 7 found that a lack of cost-effectiveness did not justify denying reimbursement to a patient who could not otherwise afford the medicine (10 October 2015). Later, Appeals Circuit/Court 5 decided that excluding the medicine from reimbursement on economic grounds is consistent with the patient’s right to health (3 November 2015). (NB: There are seven circuits/courts of appeal. Medicines claims are randomly assigned to a circuit.) The court’s decisions to reimburse expensive medicines contradicted the national rules for medicines selection and financing (i.e. Ministerial Order 86/2015), and sometimes also human rights principles in the ICESCR and General Comment No. 14.

### Part 3: access to medicines indicators in the health system ‘environment’

Only half of the expected data points were retrievable. No country reported data for all eight indicators, therefore the denominator (number of countries from which data could be retrieved) changes for each indicator reported below. Constitutional recognition for access to medicines was reported in part 1. By 2015, 123/157 countries (78%) adopted an official national medicines policy and 107/173 countries (62%) had an essential medicines lists. See Figure 2.

The average national public spending on pharmaceuticals per capita ranged from US $0.51/year ($0.00-$17.56) in low income, $4.35/year ($0.00-$17.56) in lower-middle, $16.13/year ($10.01-$71.82) in upper-middle, and $286.40/year ($2.94-$601.42) in high-income countries. To determine whether government spending was sufficient, government reports were compared to the the $12.90/capita annual minimum threshold to provide a basket of 201 essential medicines, as determined by the Lancet Commission on Essential Medicines Policies [3]. Spending was above the threshold in few low- and lower-middle income countries (except Afghanistan, Morocco, Iraq, and Tuvalu), and in most upper-middle and high-income countries (except in Gabon and the Seychelles).

**Figure 3.**
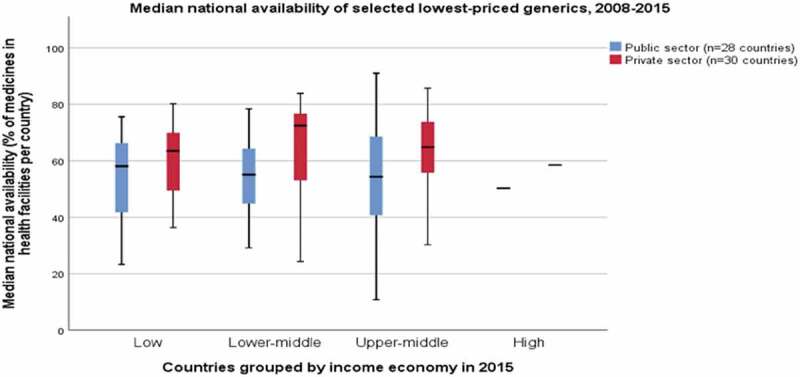
Median national availability of lowest priced generics in the public and private sectors, 2008–2015. Data source: Health Action International. Medicine Prices, Availability, Affordability and Price Components [online database] Available from: http://www.haiweb.org/medicineprices/.

The median availability of a selection of lowest-price generic medicines surveyed is slightly higher in private facilities than in public centres. See Figure 3. Median availability rarely met the 80% global target, except in a republic of Russia (both sectors) and in the private sector of Afghanistan, Tajikistan, Sudan, and Boston, USA.

National measles immunisation rates met or exceeded the 95% global coverage target to eradicate the disease, in 39% (59/153) of all countries studied (median_LICs_ = 58.5%, interquartile range (IQR) 79.5–95.0%, n_LICs_ = 12 countries; median_LMICs_ = 82.0%, IQR_LMICs_ = 59.3–94.0%, n_LMICs_ = 36; median_UMICs_ = 93.0%, IQR_UMICs_ = 81.0– 97.5%, n_UMICs_ = 53; median_HICs_ = 92.0%, IQR_HICs_ = 88.0–96.0%, n_HICs_ = 52).

National diphtheria-tetanus-pertussis immunisation rates met or exceeded the 90% global target in 43% (42/98) of all countries studied (median_LICs_ = 84%, interquartile range (IQR) 63-93%, n_LICs_ = 33 countries; median_LMICs_ = 86%, IQR_LMICs_ = 66-95%, n_LMICs_ = 44; median_UMICs_ = 91%, IQR_UMICs_ = 83-95%, n_UMICs_ = 21). Data was only available from one high income country (Equatorial Guinea, 41% DTP3 coverage).

## Discussion

This article demonstrates that human rights principles and WHO policy have been embedded in national law and policy for essential medicines and practically implemented in national health systems. Part 1 presents the first systematic, cross-national content analyses of different legal documents relevant for access to medicines, and examples of essential medicines and human rights principles in national law and policy. Some, but not all, of the 12 policy points in the analytical framework are embedded in domestic UHC law and national medicines policies. This research presents innovative ideas for embedding language promoting access to medicines in national constitutions, medicines policies and UHC legislation. National policy makers can use the example texts in this thesis (found in Annexes 1 and 2) as a starting point for designing national law and policy.

In part 2, the case study of Uruguay shows that even the most rights-compliant legislation for access to medicines (determined in part 1) is subject to the unique and inconsistent interpretation of domestic courts. The decisions of national judiciaries can diverge, sometimes dramatically, from globally-accepted interpretations of the right to health. These findings show that medicines litigation in Uruguay offers relief for some individual patients, but fails to address the structural problems behind high medicines prices. More generally, this study illustrates that both the black-and-white letter of national law, as well as domestic courts’ interpretations of that law, should be considered when determining to what degree a legal system takes a human rights approach towards essential medicines.

Part 3 reports on States’ achievement of their right to health obligations for essential medicines in 2015 at the dawn of the 2030 Agenda for Sustainable Development. Many countries for which data were available still fail to meet the official targets. These findings offer an updated reference point to measure future achievements on essential medicines as part of the right to health under the SDGs. The challenges of monitoring access to medicines globally, highlighted during the tenure of the Millennium Development Goals, regrettably persist in the era of Sustainable Development [24,25]. This monitoring study helps move modern human rights practice beyond the tradition of ‘naming, shaming, and litigating’ rights violations towards real-time measuring and monitoring rights realisation [57].

### Theory and practice: the effect of national law and policy on access to medicines

To the author’s knowledge, this thesis is currently the most robust academic endeavour to develop the evidence base to study the effect of national law and policy on access to medicines in LMICs. A large body of public health law implementation and evaluation research exists, albeit mostly in the US context [28,58,59,60,61,62]. These studies are often based on reliable online repositories of legislation and policy in English, implementation mechanisms described in scholarship and understood in practice, and robust datasets of outcome measures- all of which are commonly unavailable or underdeveloped in LMIC contexts.

One of the most significant contributions this thesis makes is to map and critically analyse different types of national legislation and policies related to access to medicines in LMICs, where there was previously little to no data readily available for analysis. Importantly, this ‘policy mapping’ exercise used transparent and reproducible methods, while coding and describing the 12 policy measures being studied, providing data for future research. As suggested by Scott Burris and colleagues, future studies can use the legislation and policy presented in this article as an outcome of policymaking studies (i.e. to understand the determinants of the policymaking process) as well as an independent variable in evaluation research (i.e. to examine the impact of law) [28]. This thesis critically analysed the content of national law and policy using a framework that is comparable across jurisdictions, types of legal instruments, and time.

Part 2 of this study examines lawmaking by judges. This study has shown that how judges understand international human rights law, and specifically the right to health, has important implications for how national law will be interpreted and, ultimately, whether patients will receive government-financed medicines.

The eight indicators of access to medicines in part 3 offer useful insight into their feasibility as dependent variables in future evaluation studies examining the impact of national law and policy.This study also demonstrates that reliable data from national health systems- a crucial ingredient for evaluation studies- is scarce in many countries. The question also arises of whether these eight indicators are the best proxy measures of changes in access to medicines in health systems. Further discussion about suitable indicators to evaluate access to medicines is available elsewhere [63].

### Future research

One question not addressed by this study but worthy of future research is: *what effect do rights-based legal provisions in national law and policy have on access to medicines in health systems and on broader population health outcomes?* Scott Burris notes that comparative policy analysis in the context of public health is under-theorised yet it has a rich diversity of policy aspects under study, implementation processes, and outcomes [29]. The field of access to medicines is no different. Implementation research is essential to understand how law and policy for access to medicines is translated into desired results, both in terms of the health system (i.e. financing and availability of medicines) and population health outcomes (i.e. morbidity and mortality rates). Piecemeal evidence suggests that a constitutional right to health may shape the ‘institutional environment’, leading to increased and better health service delivery, and it may lead to increased public spending on healthcare [64,65]. However, there is need for compelling and authoritative studies confirming a causal relationship between a constitutional/legal right to health, changes in the health system environment, and positive health outcomes. Research should also investigate the mechanisms by which national law and policy achieve intermediate outcomes (i.e. better access to medicines) and population-level impacts.

### Impact on policy

Currently, WHO’s *Guide for developing and implementing a national medicines policy* (2001) omits any mention of a government obligation to provide essential medicines to those who can not provide for themselves (a cornerstone of the right to health and the UHC concept). Moreover, WHO does not have any specific guidance for Member States to write or reform national legislation promoting universal access to medicines in UHC schemes, which is currently the subject of high-level political declarations and dominant debate in global health policy. In light of these gaps, WHO should develop modern guidance documents for the UHC era using the 12-point policy checklist and examples of legal text from this thesis as a starting point. Some of these examples translate the recommendations of the WHO Consultative Group on Equity and UHC for making fair choices on the path to UHC into legal provisions for national law [10]. This thesis may also assist WHO to develop model legislation for medicines reimbursement, which is goal 7 of WHO’s 2016–2030 Medicines & Health Products Strategic Programme [11].

WHO should establish an online repository of national health law in order to centralise the results of these legal mapping exercises. In this repository, legal texts in their original language and translations in English or other UN language can be deposited and publicly consulted. To enhance human rights monitoring and reporting, Member States should self-report on two principles for access to medicines when they deposit legislation: 1) government financing for medicines for the poor and vulnerable groups, and 2) measures to control medicines prices (i.e. prioritising medicines reimbursement based on cost-effectiveness, use of pricing policies and/or TRIPS Flexibilities). Indicator 1 ensures a government duty to provide essential medicines to those who can not provide for themselves- the crux of ‘core obligations’ under the right to health. Indicator 2 can help set objective boundaries to the right to health, and protect against unreasonable patient requests and spurious litigation for high priced medicines. A concise self-report is a quick snapshot of legal provisions; it is less laborious than an entire country profile and can be verified by consulting the laws in the repository.

National policy makers from the executive and legislative branches can preform their own monitoring exercise using the 12-point policy checklist to evaluate national law and policy for medicines. This exercise can identify gaps or weaknesses in existing law for improvement through future reform. The example legal texts in this thesis can be a source of inspiration for legislators writing or amending national law or policy. Moreover, national policy makers should adopt and finance a monitoring and reporting plan for indicators of access to medicines in line with the recommendations in this study and from the Lancet Commission for Essential Medicines Policies. Areas of deficiency should lead to a documented plan for improvements within a set timeframe.

Domestic judges should familiarise themselves with the right to health and its interpretation in international human rights law, especially the concepts of essential medicines selection and progressive realisation (i.e. there is no immediate right to all available treatments but a duty to continuously and gradually expand access) [66]. Most importantly, domestic judges should interrogate whether the government has used a maximum of its available resources to provide the medicine(s) in question (part of the standard of reasonableness) before ordering the public to pay for it [14]. Such a reasonableness test should lead to enhanced government action to reduce medicines prices and more efficient use of public resources to finance medicines.

### Strengths and limitations

This thesis is the first systematic enquiry into national law and policy for access to medicines in LMICs. Data collection drew from reputable repositories of constitutional and case law, and secondary datasets from Health Action International’s medicines pricing database and WHO/UNICEF, among others. National medicines policies and health legislation were collected using systematic online search and crowdsourcing methods, and a data extraction template. UHC legislation was collected from a purposive sample of 16 countries, which is the largest known comparison of UHC legislation from LMICs. These steps minimised the risk of a reporting bias. In summary, this study has assembled the most comprehensive collection of full-text national medicines policies and domestic health legislation for medicines from LMICs to date.

Little data for indicators of access to medicines was available for *government financing* and *medicines availability* in both sectors, and for countries in certain economic categories. Compared to the 2008 report of these indicators, this thesis uses the same methodology (in as far as possible) and reports data from more countries.

One significant strength of this study is its reliance on primary sources of national law and policy, which are more objective than the common practice of reporting key informants’ own interpretations of legislation and policy. Using national law and policy in its original language raises the question of correct translation to English and interpretation within the local legal context. To address these issues, multi-lingual and multidisciplinary research teams trained in law and medicine collected national laws and policies, and then extracted, translated and analysed relevant legal provisions. To ensure the correct interpretation of national law and policies, this study used an analytical framework based on global standards in WHO’s essential medicines policies and international human rights law. These global standards establish clear, standard definitions and legal concepts that have the greatest chance of being consistent over time and across policy/legal instruments. Therefore, unless otherwise stated in the definitions section of national law and policy, it was reasonably assumed that the terminology and concepts unique to these global standards are broadly understood in the same way in national law and policy. Second, at least one national pharmaceutical policy expert per country (except for Algeria and Nigeria) was consulted to confirm that all relevant national law and medicines policies had been located and accurately understood.

## Conclusion

International human rights law imparts important principles that are commonly embedded in the text of national law and policy for access to medicines and traceable through proxy indicators in health systems. This thesis offers researchers and policy makers the tools and the examples to translate human rights law and WHO’s essential medicines policies into national legal commitments and to monitor government actions for universal access to essential medicines as part of SDG Target 3.8. It can also inform WHO’s future guidance on UHC and essential medicines. This thesis assembled the essential building blocks to study and generated hypotheses to test the effect of national law and policy on access to medicines and population health in LMICs.

## Data Availability

The country case studies and data referred to in this article can be found on the project page: http://www.healthandgender.org/accesstomedicines.html. The data that support the findings in part 3 are available in Figshare at https://figshare.com/s/8cda5bd90602faa5ac9e. These data were derived from the following resources available in the public domain: The Constitute Project (https://www.constituteproject.org/content/about?lang=en); Health Action International’s Medicine Prices, Availability, Affordability & Price Components online database (http://www.haiweb.org/medicineprices/); WHO Development of Country Profiles and moni- toring of the pharmaceutical situation in countries (http://www.who.int/medicines/areas/coordination/coordination_assessment/en/); WHO Essential Medicines and Health Products Information Portal (http://apps.who.int/medicinedocs/en/); WHO & UNICEF coverage of DTP3 online database (http://apps.who.int/immunization_monitoring/globalsummary/timeseries/tswucoveragedtp3.html); WHO vaccine-preventable diseases monitoring system (http://apps.who.int/immunization_monitoring/globalsummary); OECD Health at a Glance 2009 – Pharma- ceutical expenditure (http://dx.doi.org/10.1787/health_glance-2009-en); OECD Health at a Glance 2015 – Financing of pharmaceutical expenditure (goo.gl/xHuXG6).

## Paper context

Access to essential medicines is a crucial aspect of the right to health and the SDG Target 3.8 for universal health coverage. Few studies examine how national law and policy are written to support universal access to essential medicines. This article reports that human rights principles and WHO policy are embedded in national law and policy related to essential medicines, and practically implemented in health systems. WHO should develop guidance for national legal/policy reform promoting access to medicines.
